# Privacy Beyond the Face: Assessing Gait Privacy Through Realistic Anonymization in Industrial Monitoring

**DOI:** 10.3390/s26010187

**Published:** 2025-12-27

**Authors:** Sarah Weiß, Christopher Bonenberger, Tobias Niedermaier, Maik Knof, Markus Schneider

**Affiliations:** Institute for Artificial Intelligence, Ravensburg-Weingarten University, Leibnizstraße 15, D-88250 Weingarten, Germany

**Keywords:** anonymization, domain adaptation, gait recognition, Industry 4.0, pose estimation, privacy, re-identification

## Abstract

In modern industrial environments, camera-based monitoring is essential for workflow optimization, safety, and process control, yet it raises significant privacy concerns when people are recorded. Realistic full-body anonymization offers a potential solution by obscuring visual identity while preserving information needed for automated analysis. Whether such methods also conceal biometric traits from human pose and gait remains uncertain, although these biomarkers enable person identification without appearance cues. This study investigates the impact of full-body anonymization on gait-related identity recognition using DeepPrivacy2 and a custom CCTV-like industrial dataset comprising original and anonymized sequences. This study provides the first systematic evaluation of whether pose-preserving anonymization disrupts identity-relevant gait characteristics. The analysis quantifies keypoint shifts introduced by anonymization, examines their influence on downstream gait-based person identification, and tests cross-domain linkability between original and anonymized recordings. Identification accuracy, domain transfer between data types, and distortions in derived pose keypoints are measured to assess anonymization effects while retaining operational utility. Findings show that anonymization removes appearance but leaves gait identity largely intact, indicating that pose-driven anonymization is insufficient for privacy protection. Effective privacy requires anonymization strategies that explicitly target gait characteristics or incorporate domain-adaptation mechanisms.

## 1. Introduction

**Figure 1:**
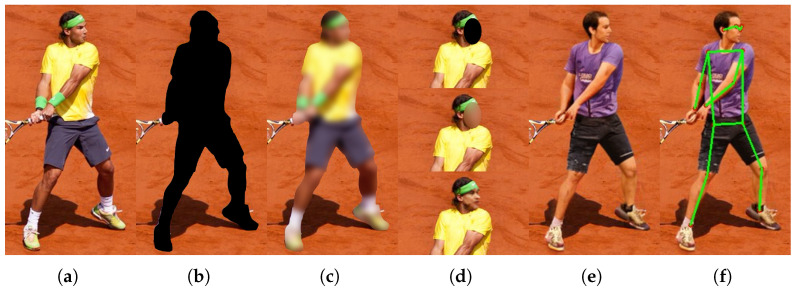
Examples for different kinds of anonymization, based on the same image from the MS COCO [4] object detection dataset. (**a**) Section of original base image; (**b**) full-body masking; (**c**) full-body blurring; (**d**) face anonymization, top to bottom: face masking, face blurring, realistic face anonymization with DeepPrivacy2 [5]; (**e**) realistic full-body anonymization with DeepPrivacy2; (**f**) human pose visualization for full-body anonymization.

Industry 4.0 environments place high demands on privacy, when camera-based systems monitor workflows, ensure safety, and optimize production [1,2]. These systems inevitably capture privacy-sensitive data, including human features that can uniquely identify a specific person. Traditional anonymization relies mainly on face blurring, face synthesis, or full-body obfuscation, examples of which are illustrated in Figure 1. Face anonymization is used most frequently, while approaches addressing full-body or appearance-based anonymization are still less common [3].

Beyond facial characteristics, human pose and gait patterns represent biometric identifiers capable of revealing identity even when the face is obscured [6]. Surveys on gait recognition establish it as a biomarker for long-distance and non-cooperative scenarios [7,8,9]. Appearance-based methods further explain how gait recognition operates using visual cues when faces are absent [10]. Occlusion-focused work provides a systematic taxonomy showing that gait remains a viable biometric even when body parts and faces are obscured by obstacles or clothing [11]. Together, these findings highlight gait as one of the most distinctive biometric traits. These factors collectively limit their applicability for systematic evaluation of anonymization, where unmodified pixel data and surveillance-relevant viewpoints are essential.

Anonymization methods differ not only in how strongly they suppress visual appearance, but also in whether they preserve the structural representation of the human body. This study considers anonymization that replaces visual appearance while maintaining the overall human structure, such that motion patterns remain observable. The analysis is conducted as a proof of principle under these conditions, assessing whether identity-related information encoded in motion persists despite the removal of visual appearance cues.

Beyond traditional anonymization techniques, advanced methods generate synthetic full-body replacements instead of obfuscating or shifting the original pixels. Realistic full-body anonymization, as illustrated in Figure 1e, introduces an unexplored privacy risk: despite synthetic appearance, pose and motion trajectories remain structurally linked to the original individual. This raises the question of whether gait-based identity cues persist after such pose-driven anonymization. Existing work provides no metric to quantify what magnitude of keypoint displacement is required to disrupt gait sufficiently for recognition algorithms to fail, and recent analyses show that robustness of skeleton-based gait recognition under structural perturbations remains insufficiently understood [12,13,14]. This study is the first to explicitly address this problem and to investigate whether gait remains intact to a degree that enables identity linkage across original and anonymized domains through systematic experimentation.

Records of a person walking alone are enough to raise valid privacy concerns, as gait can be exploited without the target’s consent. Even at a distance and without cooperation, records can provide sufficient data for gait-based recognition [15]. This enables identification in public and private spaces, as gait does not require physical contact or active participation and is measurable from common surveillance footage [16]. Consequently, gait recognition presents unique challenges for privacy protection, as individuals may be unaware that such biometric processing is taking place.

However, studying the privacy risks of gait requires datasets that provide both full visual identity and gait-relevant motion cues, which existing resources only partially satisfy. Public gait datasets such as CASIA [17] and GRIDDS [18] offer large-scale pose- or silhouette-based benchmarks, yet not all include original, unaltered RGB sequences. Their viewpoints are restricted to canonical side views, whereas real-world surveillance employs oblique or top-down CCTV viewpoints. Datasets that adopt a CCTV perspective, such as CCPG [19], often contain pre-obfuscated faces, preventing an assessment of anonymization effects on full-body appearance. In addition, several datasets require formal licensing agreements that can conflict with industrial compliance policies, and some older collections no longer process access requests. These constraints limit their suitability for systematically analyzing how anonymization alters biometric information.

Further, privacy regulations, such as the European GDPR [20], the Canadian PIPEDA [21], and the South Korean PIPA [22], demand safeguards when processing such data. Anonymization offers a promising way to enable legally compliant monitoring, since it can reduce or remove personal identifiers while still allowing data use. However, traditional methods (e.g., blurring, masking) often degrade information that may be required for downstream tasks [23], while still leaving certain biometric cues intact [24]. For example, masking can obscure carried objects, and face blurring does not alter gait, leaving identity partly exposed. In regulated industrial environments, an anonymization method is therefore considered insufficient whenever identity linkage through gait remains possible.

Traditional anonymization approaches, such as blurring and pixelation are widely applied for their simplicity but often ineffective for specific tasks. Beyond information loss, they have shown to be reversible [25,26,27]. Anonymization strategies, such as [28], are able to remove individuals entirely from frames and only supplies their pose. Although this ensures privacy guarantees, the resulting heavily altered images have limited applicability for downstream analysis.

In contrast, realistic anonymization methods generate synthetic replacements that aim to be natural-looking and can be even context preserving, allowing continued use of the data. These methods are more sophisticated but still limited, with only a few focusing on realistic full-body anonymization, such as the work of Brkic et al. [29] and Hukkelås et al. with DeepPrivacy2 [5]. More recently, the diffusion-based approach of Zwick et al., FADM (Full Body Anonymization using Diffusion Models) [30], was proposed, enabling context-aware anonymization that better preserves downstream utility. An example of realistic full-body methods is shown in Figure 1. As these methods preserve both pose and global scene consistency, they form the most relevant test case for evaluating residual biometric leakage beyond visual appearance.

While anonymization is essential for privacy compliance, its integration into vision pipelines is not without consequences. Several studies have demonstrated performance drops in core computer vision tasks when anonymized data are used. In an additional study, Hukkelås et al. [31] reported a sharp decline in object detection, segmentation, and pose estimation when applying both traditional and realistic anonymization. Traditional methods such as full-body blurring or pixelation caused severe degradation. Blurred pedestrians were almost entirely undetectable in instance segmentation tasks. The realistic approach preserved performance more effectively but still introduced a consistent accuracy loss across all tasks.

Triess et al. [32] show that full-body pixelation renders individuals essentially invisible to pose estimation and action recognition systems. Refs. [33,34] further confirm this trend, finding that anonymization reduces model accuracy, though the extent depends on the method and task. These results emphasize that anonymization not only protects privacy but also reshapes the data in ways that impact downstream learning.

Earlier work by the author [35] expanded this by analyzing how anonymization altered the learning process of models compared to training on unaltered data. Further, the influence of anonymization to classes co-occurring with anonymized individuals was investigated. The results demonstrated that image-level modifications through anonymization propagated into training, leading to measurable shifts in feature representations and inference accuracy. This provided a systematic methodology to evaluate not only performance losses but also the mechanisms by which anonymization influenced the model.

Building on this foundation, the present study investigates whether realistic full-body anonymization can conceal identity beyond visual appearance. Specifically, it focuses on the effects of gait as a robust biometric marker. This focus is motivated by the growing dependence on monitoring in Industry 4.0, where operational safety and efficiency must be balanced with legal and ethical obligations to protect individual privacy. Similar requirements apply to healthcare, where long-term observation of individuals is necessary, but personal identity must remain protected.

To date, no study has systematically tested whether realistic anonymization removes gait-based identity cues, despite preserving the original pose. The authors of DeepPrivacy2 explicitly acknowledge this risk, noting that their DensePose-based anonymization might retain pose information, which has the potential for gait-based identification. Triess et al. [32] showed that DeepPrivacy2 maintained pose recognition and action recognition capabilities, but they did not evaluate its impact on person identification via gait. Recent work such as GaitGuard [36] reflects a growing awareness of gait anonymization needs, but their presented method mostly keeps the visual appearance of the individual.

This research therefore aims to close this gap. We extend existing evaluation methodologies of [35] to address gait-related questions raised by Hukkelås et al. [5]. We further extend the findings of Triess et al. [32] to additional downstream tasks. Specifically, the study assesses whether realistic full-body anonymization, while obscuring visual identity, also alters gait. By comparing the performance of algorithms trained on original versus anonymized data, the study evaluates if anonymization effectively disrupts gait-based identification while retaining utility for intended applications.

The novelty of this study lies in systematically evaluating whether realistic, pose-preserving anonymization disrupts identity-relevant gait characteristics. The analysis quantifies keypoint shifts introduced by anonymization, examines their effect on downstream gait-based person identification, and tests whether anonymized sequences remain linkable to identities in unaltered recordings. As no established metric describes how much pose distortion is required to invalidate gait recognition, controlled experiments tailored to this question are essential. This work provides the first such systematic evaluation and applies it to realistic full-body anonymization.

## 2. Methods and Materials

The following methodology establishes a setup to assess how realistic full-body anonymization alters pose quality and downstream gait recognition. The approach integrates a custom-recorded dataset, pose extraction from original and anonymized frames, and the pose-based gait recognition framework of FastPoseGait [37] with GPGait++ [38]. Additional analyses quantify keypoint deviations introduced by anonymization and their influence on pose-based re-identification through gait. Two model variants, trained on original and anonymized poses, are evaluated under matched open- and mixed-set conditions. This setup enables systematic examination of training behavior, cross-domain generalization, and sensitivity to anonymization-induced pose changes.

### 2.1. Pose-Based Gait Recognition

FastPoseGait is a toolbox and benchmark for pose-based gait recognition, able to identify and re-identify individuals from sequences of human keypoints rather than silhouettes or visual appearance. The framework compares identity embeddings across samples, enabling both person identification or re-identification. The distinction arises from the evaluation protocol: identification classifies a gait sequence among known identities (closed set), while re-identification matches a sequence to gallery embeddings (open set).

The framework supports multiple pose-based models to learn general discriminative gait representations that distinguish between identities of walking sequences. The latest addition, GPGait++, explicitly targets generalization across unseen domains, which is a known limitation of prior methods such as GaitGraph2 [39], GaitTR [40], and GPGait [41]. This focus aligns with real-world scenarios, where domain shifts and environmental variability remain critical challenges for practical gait recognition applications. For the experiments, the FastPoseGait framework is configured to train and evaluate multiple GPGait++ models as gait recognition systems for input poses. Details about the training and evaluation process are supplied in Section 2.3.

### 2.2. Dataset

Due to the absence of publicly available unanonymized images within pose-based gait datasets with a CCTV-viewpoint in an industrial setting, an internal dataset was recorded. The chosen recording configuration reflects a common ceiling-mounted CCTV-like perspective used in industrial and healthcare environments and provides a reproducible reference scenario that can be directly integrated into existing surveillance installations. Accordingly, the dataset is not intended to model all possible deployment geometries but serves as a controlled setup to examine whether gait-related identity information persists under realistic full-body anonymization. A single fixed camera viewpoint was deliberately used to mirror the established gait benchmark CASIA [17], while avoiding the complexity of multi-camera calibration.

Recordings were conducted in a controlled indoor environment with a mix of artificial and natural lighting conditions and static backgrounds, reflecting typical industrial and healthcare surveillance settings. The recorded scenes and gait sequences followed the design principles of the CASIA dataset. Data collection involved ten participants aged between 20 and 35 (no known motion related impairments, 8 male, 2 female) and covered six scene types: normal walking (two separate runs), carrying a light object, carrying a heavy object, walking with changed clothing, and walking in cluttered or chaotic backgrounds. Each scene included eleven walking sequences captured from a fixed CCTV-like viewpoint (camera height 2.8 m, tilt angle 20° downward). To maximize the quantity of collected data under a single-camera constraint, each participant performed multiple walking sequences while the starting orientation was systematically varied. Subjects walked away from the camera, with starting positions incrementally shifted counter-clockwise in 18° steps until walking directly towards it. This design increased viewpoint diversity while preserving a consistent camera geometry. Recordings were performed using a single Azure Kinect as RGB camera at 30 FPS and 720p with ROS2. Camera-intrinsic parameters supplied by the manufacturer were used to correct lens distortion in the recorded frames.

**Figure 2:**
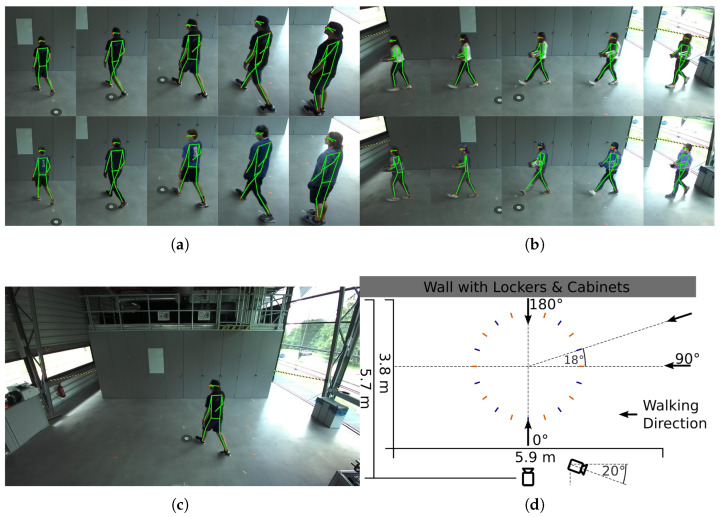
Top: Examples of recorded data for human poses in original (top line) and anonymized (bottom line) images. (**a**) Normal walking scene. (**b**) Carrying object scene. Bottom: (**c**) Full view of the recorded scene. (**d**) Recording setup.

All image sequences were anonymized using DeepPrivacy2, employing default configuration settings. The anonymization was performed with a fixed seed (seed =0) and identity tracking enabled (track = true). Using an identical seed ensured that DeepPrivacy2 produced reproducible anonymized images given the same input frames and model weights. The recommended full-body configuration (FB_cse), combining pose guidance with a segmentation mask, was used throughout. Human poses were extracted using the YOLOv11m-Pose model on both the original and the anonymized frames. To ensure consistent comparison between corresponding sequences, only frames with poses detected in both the original and anonymized images were retained. Specifically, for each pair, undetected frames were discarded, and only the longest continuous subsequence of matching frames was kept. This guaranteed alignment and identical frame counts between the original and anonymized pose sequences. Figure 2 presents representative dataset samples and illustrates the recording setup.

### 2.3. Experiments

Our experiments investigate the influence of anonymization on pose-based gait recognition. They analyze structural pose deviations introduced by anonymization and assess the downstream impact on model training and evaluation. By comparing models trained on original versus anonymized poses under both open- and closed-set conditions, the experiments quantify how anonymization affects recognition performance and generalization across identities and environments.

#### 2.3.1. Pose Differences Between Original and Anonymized Images

Poses were extracted from both original and anonymized images, and corresponding keypoints were compared frame by frame. The YOLOv11 Pose model predicted 17 keypoints per person, covering major body joints such as the head, shoulders, elbows, hips, knees, and ankles. The model produced consistent keypoints across repeated runs on the same image, ensuring that observed differences originated from image modifications rather than inference randomness. For each frame, the positional difference between the same keypoint in the original and anonymized image was computed, e.g., the total difference between the left hip keypoint of the original image and the anonymized one of the same frame was calculated. These differences were averaged per keypoint across all sequences and subjects, providing a quantitative measure of pose distortion introduced by anonymization. This analysis enabled the assessment of the extent to which anonymization altered the geometric consistency of human poses.

#### 2.3.2. Training and Evaluation

Following the methodology of [35], two models were trained: one using poses derived from original data (ORG) and one using poses from anonymized data (ANON). Each model was subsequently evaluated on both the original and anonymized evaluation sets (on ORG, on ANON), e.g., an evaluation with a model trained on original but evaluated on anonymized data was called ORG on ANON.

As commonly adopted in gait recognition studies, accuracy was used as the main evaluation metric, quantifying the fraction of correctly assigned identity labels [38,39,42]. Because each probe sequence produced exactly one prediction, it reflected how reliably the model retrieved an identity from the gallery. This is also known as a rank-1 evaluation in gait benchmarks and directly assesses the discriminative strength of the learned gait representation without relying on ranking lists.

Training adopted the default GPGait++ configuration and parameters of FastPoseGait unless stated otherwise. Only a small set of parameters was adjusted to match the characteristics of the custom dataset: the number of identity classes was reduced to reflect the available training IDs (num_class 7), and the sampler operated with fewer samples per identity (batch_size [4, 5], [IDs per batch, samples per ID]). Due to the smaller number of recorded identities compared to standard gait datasets, the total number of training iterations was reduced to 25,000 (for reference, the default GPGait++ configuration on CCPG employs 100 identities with 40,000 iterations). Throughout training, the loss was continuously monitored to track convergence behavior and detect potential overfitting. To further ensure training stability and analyze model progression, checkpoints were saved every 1000 iterations and subsequently evaluated to determine the iteration range yielding stable and representative results. All remaining settings followed the GPGait++ defaults. Both training and evaluation procedures were conducted using an NVIDIA A40 GPU with 48 GB VRAM.

Two experimental configurations were applied. In the open-set setup (new persons), a subset of identities was used for training across all scenes, and evaluation was performed on previously unseen identities to assess generalization to unfamiliar individuals under known conditions. In the mixed-set setup (new situations, known and unknown IDs), a subset of identities was used for training on a subset of scenes, and evaluation was conducted on unseen scenes for both known and unknown identities, enabling the analysis of model robustness to environmental and contextual variation. The specific ID and scene splits are summarized in Table 1. We aimed for a 70/30-split for training and evaluation. Due to differences in scene length and walking speeds of individuals, this was not always possible for the defined experimental setup.

## 3. Results

First, keypoint shifts between original and anonymized frames are measured to characterize pose distortions relevant for gait. Subsequently, pose-based gait recognition performance is reported for open-set and mixed-set configurations, comparing models trained on original versus anonymized poses under matched training conditions. The analyses focus on convergence behavior, domain transfer between original and anonymized data, and the influence of scene context such as object carrying and clothing changes on recognition accuracy.

### 3.1. Regarding Pose Differences Between Original and Anonymized Images

To quantify how anonymization affects pose estimation, keypoint positions of the original and anonymized images were compared. Table 2 summarizes the positional shifts per joint. The keypoint comparison reveals that anonymization alters poses non-uniformly. Head-related points (nose, eyes, ears) remained relatively stable with mean shifts below 5 pixels, while the largest displacements occurred at extremities such as elbows, wrists, knees, and ankles (up to ≈13 pixels). The displacement increased with distance from the body center, indicating that minor central inaccuracies propagated along the limbs.

Right-side joints underwent slightly larger deviations than their left counterparts, caused by the camera perspective and walking direction, which mainly exposed the left side and partially occluded the right body side. Therefore, pose estimation on anonymized frames showed reduced stability for right-side joints, amplifying errors for body parts farther from the camera. This view-dependent effect demonstrates that anonymization interacts with viewpoint geometry.

Lower-body joints (hips, knees, ankles) experienced strong shifts of five to nine pixels, directly affecting stride- and rhythm-related geometry. The cumulative displacement from hips to ankles suggests that anonymization mainly perturbs limb geometry and motion cues, while upper-body alignment remains stable. As gait recognition depends on precise joint movements over time and space, these distortions reduce the consistency of pose sequences and can change the features the model learns to recognize gait patterns.

When models were trained and evaluated across different data types (original and anonymized), variations in joint positions likely hindered model adaptation, leading to uneven performance and lower accuracy across datasets. Overall, anonymization introduced measurable geometric distortions, most prominent at motion-critical joints, with possible impacts on both pose stability and gait-based model generalization.

### 3.2. Open Set

**Figure 3:**
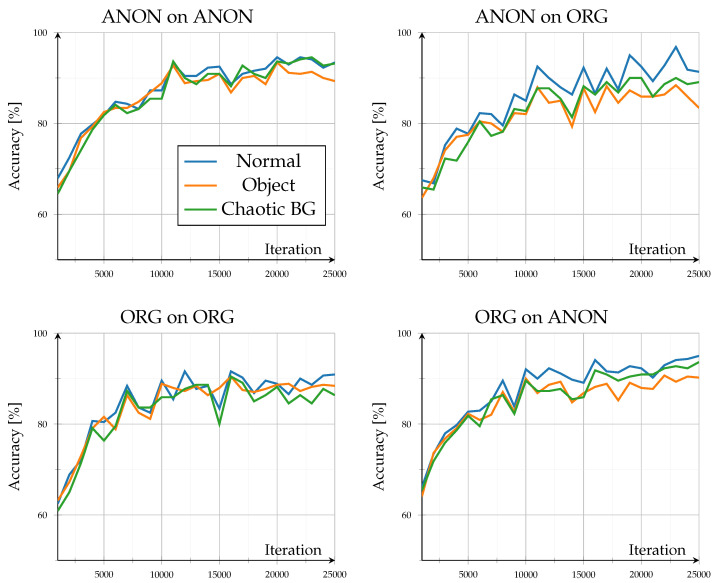
Open-set results: results for accuracy (%) of unknown identities for known situations. Showing all different model and evaluation combinations.

The open-set evaluation investigated how models generalized to unseen identities. Across all configurations, performance saturated beyond roughly 20,000 iterations, demonstrating stable convergence without overfitting (see Figure 3). Accuracy on unseen data remained high, ranging between 83.4% and 95% after 25,000 iterations, consistent with the results reported by the authors of GPGait++ for comparable datasets. Scene-specific variations stayed within ±2% throughout training, indicating that background complexity and object interaction exerted only minor influence in both data domains. Overall trends showed minimal differences: training and testing on the same data type (Org on ORG, ANON on ANON) led to lower inter-scene variance, whereas cross-type evaluations (Org on Anon, ANON on ORG) exhibited a slightly higher variance.

**Figure 4:**
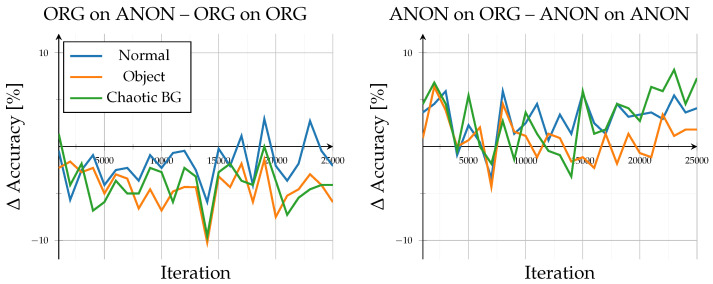
Open-set results: Difference in accuracy when comparing open-set results across domains. Left subplot shows accuracy of the ORG model evaluated on anonymized data minus the base evaluation of the ORG model on original data. Right subplot shows accuracy of the ANON model evaluated on original data minus the base evaluation of the ANON model on anonymized data.

A detailed comparison of evaluation data exchange, as in Figure 4, provides further insight into model generalization. When switching the evaluation data type (Org on Anon–Org on ORG; ANON on ORG–ANON on ANON), the model trained on original data exhibited a consistent performance decline, with accuracy reductions between −2% and −5.9% at 25,000 iterations and dropped up to −10% around iteration 14,000. In contrast, the model trained on anonymized data performed better when evaluated on original data, showing accuracy gains of approximately 1.8% to 7.3%. However, this configuration displayed stronger fluctuations, alternating between positive and negative changes, indicating less stable adaptation behavior across data domains.

Despite cross-domain discrepancies, accuracy consistently exceeded 80%, indicating that anonymization preserved essential gait structures. Overall, the results confirm the high robustness of pose-based gait recognition under realistic anonymization, showing moderate but asymmetric domain transfer effects and minimal sensitivity to environmental context.

As this open-set configuration assessed unseen identities under known scene conditions, the consistently high accuracies demonstrate strong cross-subject generalization. The model captured person-specific motion and pose characteristics rather than memorizing training identities, indicating that both original and anonymized pose representations preserved identity-discriminative information. The minimal difference between Org on ORG and ANON on ANON configurations further confirms that anonymization did not alter the fundamental gait dynamics required for a reliable cross-identity recognition.

### 3.3. Mixed-Set

#### 3.3.1. Seen-Set (Known Identities Under New Situations)

**Figure 5:**
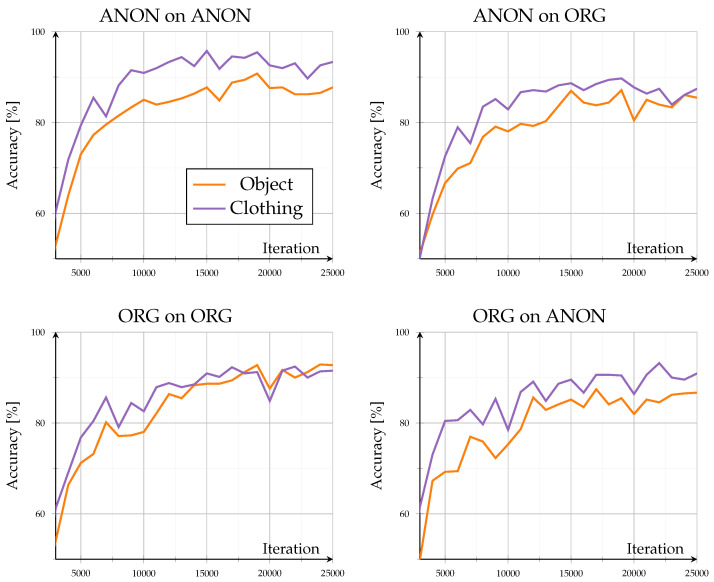
Mixed-set (subset: seen) results: results for accuracy (%) of known identities for new situations. Showing all different model and evaluation combinations.

Evaluation on known identities under new situations revealed how the model adapted to contextual variations while retaining previously learned identity representations. Across all configurations, convergence stabilized beyond approximately 20,000 iterations, with accuracies between 85.5% and 93.3% after 25,000 iterations (see Figure 5). The accuracy curves showed progression without notable oscillations, confirming stable learning behavior on familiar identities.

**Figure 6:**
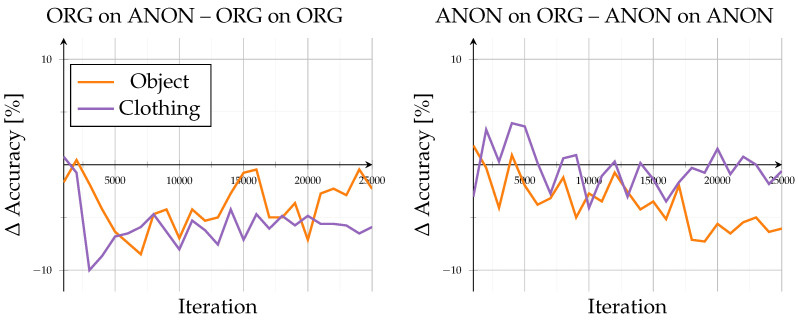
Mixed-set (subset: seen) results: Difference in accuracy when comparing mixed-set results (subset: seen) across domains. Left subplot shows accuracy of the ORG model evaluated on anonymized data minus the base evaluation of the ORG model on original data. Right subplot shows accuracy of the ANON model evaluated on original data minus the base evaluation of the ANON model on anonymized data.

Models trained and evaluated within the same domain (Org on ORG, ANON on ANON) achieved comparable accuracy, with only minor influence from scene variation. In contrast, cross-domain configurations (Org on Anon, ANON on ORG) exhibited reduced performance when switching between data types (compare Figure 6): Org on Anon resulted in moderate declines of −2.3 to −5.9% at 25,000 iterations, while ANON on ORG showed stronger fluctuations with drops between −0.6 and −6.1%.

Scene variation only had a minor influence (≤2%). Clothing-change scenes consistently outperformed object-interaction scenes by 4 to 6%, indicating that pose alterations caused by carrying objects impaired clarity more than gait alteration through changed clothing. The overall stability suggests that for known identities, anonymization mainly affects appearance rather than the structural gait representations.

#### 3.3.2. Unseen-Set (New Identities Under New Situations)

**Figure 7:**
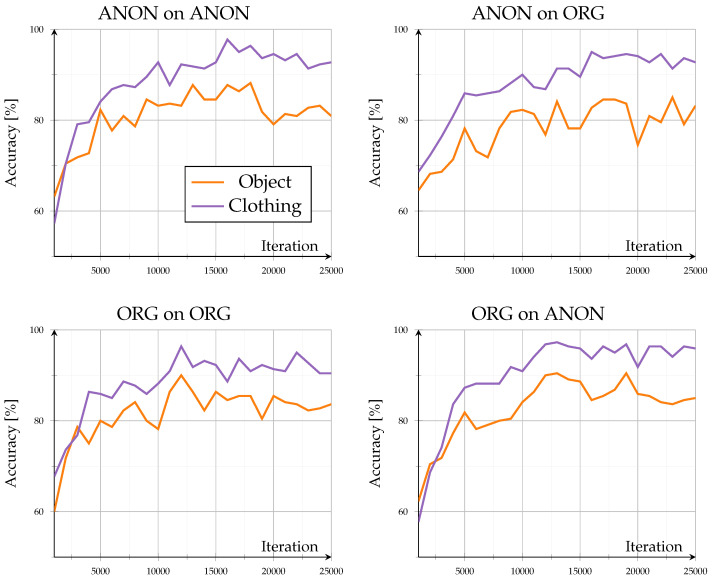
Mixed-set (subset: unseen) results: results for accuracy (%) of unknown identities for new situations. Showing all different model and evaluation combinations.

Evaluation on unseen identities under new situations assessed the model’s ability to generalize to novel subjects and contexts. All configurations reached high performance between 80.9 and 95.9% after 25,000 iterations (see Figure 7). Training saturated around 20,000 iterations, confirming stable convergence without overfitting. Scene-specific deviations remained below ±2%, indicating strong robustness to intra-scene variations, comparable to that observed for known identities.

**Figure 8:**
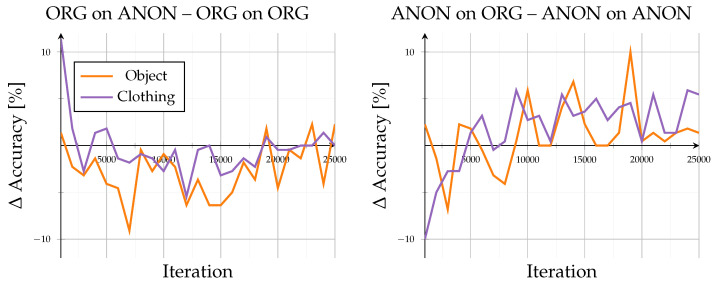
Mixed-set (subset: unseen) results: Difference in accuracy when comparing mixed-set (subset: unseen) results across domains. Left subplot shows accuracy of the ORG model evaluated on anonymized data minus the base evaluation of the ORG model on original data. Right subplot shows accuracy of the ANON model evaluated on original data minus the base evaluation of the ANON model on anonymized data.

Models trained and evaluated on identical data domains (Org on ORG, ANON on ANON) showed smooth progression from approximately 70% to 95%, flattening after about 20,000 iterations with only ±1% local fluctuations. The absence of sharp oscillations or curve crossovers indicated low variance and stable convergence within same-domain training. In contrast, cross-domain evaluations revealed distinct asymmetry (compare Figure 8): Org on Anon led to decreases between −2 and −5.9%, reaching up to −10% around 14,000 iterations, whereas ANON on ORG produced moderate positive shifts (1.8 to 7.3%) accompanied by increased oscillations and minor drops (−1 to −2%), showing higher instability than the Org-trained configuration.

The observed asymmetry suggests that training on anonymized data introduces a greater diversity in features, enhancing robustness toward real data but increasing variance. Accuracies above 80% across all conditions confirm that anonymization preserves identity-discriminative motion patterns. As evaluation occurred on previously unseen scenes, the results demonstrate strong cross-scene generalization, showing that the models maintained stable gait representations despite environmental variation, which affected performance less than the underlying gait structure.

#### 3.3.3. Combination of Seen and Unseen Set (New Situations, Known and Unknown Identities)

**Figure 9:**
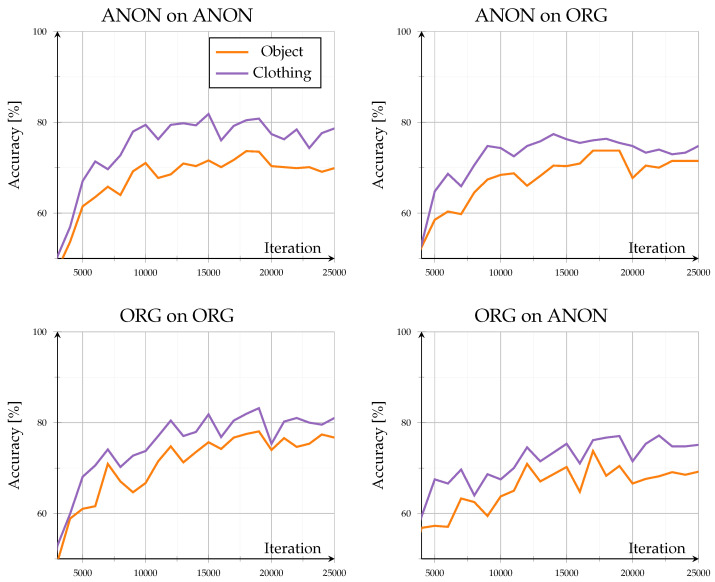
Mixed-set results: results for accuracy (%) of known and unknown identities for new situations. Showing all different model and evaluation combinations.

Evaluation on the mixed configuration of seen and unseen identities under new situations examined the joint influence of identity and scene novelty. As other experiments before, convergence remained stable beyond approximately 20,000 iterations without signs of divergence (see Figure 9). Accuracies ranged between 69.2% and 81% after 25,000 iterations. Although evaluated on the same scenes as the seen and unseen subsets, the mixed setup yielded notably lower results, indicating a compounding effect in which unfamiliar identities and altered scene conditions jointly reduced recognition stability. The decline suggests a non-linear interaction between scene and identity novelty, challenging the model’s capacity to maintain consistent feature representations across diverse conditions.

**Figure 10:**
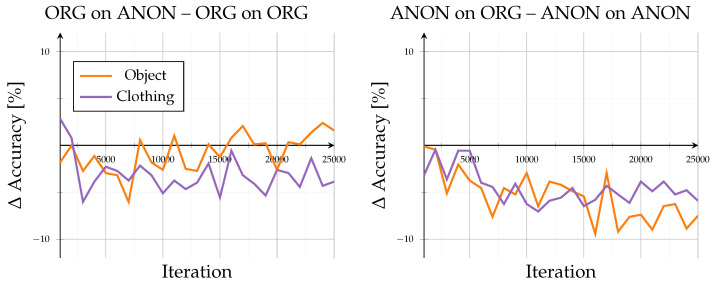
Mixed-set results: Difference in accuracy when comparing mixed-set results across domains. Left subplot shows accuracy of the ORG model evaluated on anonymized data minus the base evaluation of the ORG model on original data. Right subplot shows accuracy of the ANON model evaluated on original data minus the base evaluation of the ANON model on anonymized data.

Scene-related changes were most evident in object-interaction sequences (±6%), showing that carrying or manipulating objects increased pose variability and occlusion effects, thereby reducing stability. Clothing-change scenes remained comparatively consistent, as appearance variations influenced motion features less strongly. Domain-matched configurations (Org on ORG, ANON on ANON) yielded the highest performance, while cross-domain setups amplified oscillations due to combined domain and scene shifts. Differential analyses revealed accuracy drops under cross-evaluation (compare Figure 10): *Org on Anon* decreased by −3 to −6%, occasionally reaching −7%, with final iteration values ranging from −3.9 to 1.6%; ANON on ORG exhibited stronger declines between −5.9 and −7.5% at 25,000 iterations, with negative peaks up to −9.4%.

Compared to the open-set results, models trained on anonymized data showed higher resilience, suggesting that exposure to anonymized variability helps reduce environmental bias. The results indicate that scene variation, rather than anonymization, is the main factor limiting cross-scene generalization. Despite lower overall accuracies, all configurations stayed above roughly 70% accuracy, showing that key gait patterns remain preserved even in unseen environments.

#### 3.3.4. Combined Interpretation of the Mixed-Set

The combined interpretation summarizes overall model behavior across all mixed-set configurations, linking training stability, scene sensitivity, and domain transfer effects.

Training Stability: Across all settings, convergence was reached at approximately 20,000 iterations, confirming stable training and the absence of overfitting.

Performance per Configuration: The evaluation for IDs seen in training showed stable recognition of known identities under new situations with minimal impact from anonymization. The unseen configuration confirmed strong cross-subject generalization in new environments, indicating that gait-discriminative structures remained largely preserved. For the mixed configuration, combining known and unknown identities in unseen scenes proved most challenging, with accuracies between 69% and 81%, demonstrating that combined scene and identity novelty together reduced recognition stability.

Scene and Domain Influence: Scene changes with object interaction caused the strongest accuracy decreases, as carrying objects modified poses and introduced occlusions. Clothing-change scenes remained more stable, since appearance variation had less effect on motion features. Models trained and evaluated within the same domain (Org on ORG, ANON on ANON) showed higher stability, while cross-domain setups exhibited amplified oscillations due to domain shifts. Differential analyses revealed asymmetric transfer behavior: Org on Anon decreased by −3 to −6%, whereas ANON on ORG dropped by −5.9 to −7.5%, indicating that anonymized training led to slightly higher loss when tested on original data.

General Interpretation: Compared to the open-set results, models trained on anonymized data exhibited higher stability, indicating that increased variability in anonymized training samples enhanced adaptation to new environments. Scene variation, rather than anonymization, remained the primary factor limiting generalization across different conditions. Despite lower overall accuracies, all models achieved accuracy scores above 70%, confirming that core gait characteristics were preserved even when both identity and environment differed from the training data.

## 4. Discussion

The conducted experiments systematically assessed the influence of realistic full-body anonymization on pose-based gait recognition. Accuracies above 80% in the open- and mixed-set experiments demonstrated that essential gait-discriminative structures remained preserved despite anonymization. The following sections discuss these results regarding generalization behavior, domain transfer asymmetries, and environmental context.

### 4.1. Stability and Retention of Core Gait Patterns

The keypoint analysis showed that the observed distortions were predominantly systematic rather than stochastic, with consistent offsets across sequences and subjects and directionally stable displacements that increased with kinematic distance from the torso. As a result, the temporal structure of joint trajectories remained coherent despite anonymization, enabling gait-based models to exploit stable motion patterns.

Despite lower absolute accuracies in the mixed configuration, all models maintained accuracy values of 70% and more (80 to 90%). This consistent baseline shows that anonymization does not remove the motion patterns over time that are essential for recognizing a person’s gait. This stability demonstrates that realistic anonymization preserves functional gait information necessary for recognition tasks. However, the persistence of high accuracy for known identities also indicates that identity-specific motion characteristics remain present, suggesting that such methods anonymize visual appearance but not motion identity through gait. This observation confirms the assumption made by the authors of DeepPrivacy2 that gait-related features are likely retained for anonymization based on human pose.

### 4.2. Cross-Identity and Cross-Scene Generalization

High accuracies in both the open-set and mixed-set unseen configurations verify strong cross-subject generalization even under anonymized conditions. The preserved performance indicates that identity-discriminative motion patterns remain intact when visual appearance is replaced by synthetic body representations. This also confirms the assumption of the DeepPrivacy2 authors that gait-related information remains present after anonymization. All configurations achieved high performance between 80% and 96%, aligning with the original GPGait++ benchmarks (reported mean accuracies of 83.5% on the CASIA-B dataset). The observed training behavior further confirms that anonymization does not disrupt structural pose consistency. Hence, gait recognition on anonymized data remains reliable as long as body-joint geometry and temporal coherence are preserved.

### 4.3. Domain Transfer and Asymmetry

Cross-domain evaluations revealed asymmetric behavior between original and anony-mized data. *Org on Anon* configurations consistently decreased by approximately −3 to −6%, whereas ANON on ORG evaluations fluctuated more strongly, reaching up to 7% with increased variance. This asymmetry suggests that anonymized training introduces greater feature diversity, improving robustness toward real data but reducing stability. These findings correspond to observations for pure visual data by [5,33,35], who reported that augmented visual variability enhanced generalization at the expense of precision. Consequently, domain adaptation strategies or multi-domain fine-tuning may further mitigate this imbalance and strengthen cross-domain transfer.

### 4.4. Influence of Scene Context and Occlusion

Scene-related factors, particularly object-interaction sequences, produced the largest accuracy decrease. Carrying or manipulating objects modified local pose geometry and caused partial occlusions, which degraded recognition stability by up to ±6%. In contrast, clothing-change scenes remained comparatively stable, as they altered appearance while minorly affecting motion dynamics. This is consistent with findings of [43] that report that different clothing styles only have a limited effect on gait performance.

Our results indicate that environmental and occlusion effects, rather than anonymization, constitute the dominant limitations in cross-scene generalization. Since anonymized rendering preserves overall body topology, the observed degradation mainly originates from reduced joint visibility rather than generation artifacts.

### 4.5. Limitations

The scope of the dataset necessarily limits the extent to which the findings can be generalized. The study involved a restricted number of participants within a narrow age range, which precludes population-level conclusions. This design choice was deliberate, as the objective was a proof-of-principle analysis that isolates whether gait-based identity information persists under realistic full-body anonymization, rather than a comprehensive assessment across demographic groups.

Further, constraints about generalization were introduced through the recording configuration. All sequences were captured from a single, fixed CCTV-like viewpoint, reflecting a common industrial surveillance setup but limiting insight into how the observed effects scaled across heterogeneous camera geometries or oblique viewing angles. As a result, the reported findings should be interpreted as representative of similar surveillance configurations rather than arbitrary deployment scenarios.

In addition, the controlled nature of the recording environment reduced variability typically encountered in real-world industrial or healthcare settings. Factors such as diverse backgrounds, clothing styles, crowd interactions, and long-term behavioral changes were not fully represented. While this controlled setup enabled a focused analysis of anonymization-induced effects, it did not capture the full complexity of operational deployments.

Our analysis also assumed relatively stable sensing conditions. Pose estimation was performed on RGB data acquired under consistent lighting and motion conditions, and increased sensor noise, motion blur, or partial occlusions may interact with anonymization-induced distortions in ways not observed in the present experiments. Such effects could further influence pose stability and, consequently, gait-based recognition performance.

Finally, the evaluation was tied to a specific sensing and processing pipeline, including a single RGB camera, a particular pose estimation model, and one representative realistic anonymization approach. Although these components reflect widely used configurations, different sensors, pose extractors, or anonymization strategies may lead to quantitatively different outcomes.

## 5. Conclusions

This study investigated whether realistic full-body anonymization based on pose removed identity cues contained in human gait. Taken together, the findings demonstrate that realistic full-body anonymization suppresses appearance but preserves motion-based identity, revealing a fundamental privacy limitation previously unquantified. The results provide the first systematic evidence that pose-preserving anonymization does not break gait identity, addressing an open question left by earlier work on skeleton robustness and motion perturbations. This investigation also delivers the first quantitative analysis of anonymization-induced keypoint shifts and their direct impact on downstream gait recognition performance. By establishing a systematic evaluation framework across original and anonymized domains, the study closes a gap where no assessment protocol or distortion threshold previously existed.

The pose comparison revealed that anonymization introduced systematic but moderate geometric distortions, with small shifts at the head and larger displacements at joints located farther from the torso. Lower-body keypoints relevant for gait showed mean shifts, while overall body topology remained intact. These distortions reduced pose stability but did not alter the underlying motion patterns that enabled gait-based discrimination, indicating that appearance-level anonymization leaves the biometric signal intact.

Across all open-set and mixed-set configurations, pose-based gait recognition with GPGait++ maintained high accuracies. Models trained and evaluated within the same domain (Org on ORG, ANON on ANON) reached comparable performance, indicating that anonymized poses retained sufficient identity-discriminative information for reliable recognition. Cross-domain evaluations exposed an asymmetric domain transfer: Org on Anon consistently reduced accuracy, whereas ANON on ORG yielded moderate gains but with increased variance. Training on anonymized data therefore introduces greater feature diversity and improves robustness to real data, at the cost of higher instability.

Scene-related variation affected performance more strongly than anonymization itself. Object-interaction sequences, which induced occlusions and local pose perturbations, caused the largest accuracy drops, while clothing-change scenes remained comparatively robust, consistent with reports of limited influence of typical clothing shapes on walking mechanics. Overall, the results indicate that realistic full-body anonymization in its current form anonymizes appearance but not gait identity: core motion patterns and cross-identity discriminability are preserved. For privacy-critical applications, this implies that this anonymization alone is insufficient to neutralize gait as a biometric and must be complemented by gait-targeted obfuscation or domain-adaptation mechanisms.

## 6. Future Work

Future work on visual full-body anonymization should focus on mechanisms that explicitly target motion identity. Since performance degradation primarily arises from occlusions and reduced joint visibility, occlusion-aware pose refinement, 3D joint completion, or temporal keypoint smoothing may further stabilize anonymized pose sequences.

Extending the evaluation to heterogeneous camera placements, including wall-mounted and oblique viewpoints, provides an important direction for further work and allows clearer separation of viewpoint-related variability and anonymization-induced effects across realistic deployment scenarios.

Beyond viewpoint diversity, scaling the evaluation to larger and more diverse participant populations is essential to assess generalization across age ranges, body types, clothing styles, and long-term behavioral variability. Such extensions would enable a more comprehensive understanding of how anonymization-induced pose distortions interact with population-level gait diversity.

Progress also depends on dedicated anonymization-aware gait datasets that provide paired original–anonymized recordings, environment metadata, and standardized protocols for evaluating motion identity leakage.

Finally, integrating multimodal sensing and testing additional anonymization methods offer a promising direction for privacy-by-design monitoring solutions. Advancements in these areas will support anonymization strategies that preserve downstream utility while mitigating risks associated with gait-based identity leakage.

## Figures and Tables

**Table 1:** Data splits used for training and evaluation for the open set (unknown IDs, known situations) and mixed set (new situations, known and unknown IDs). Due to different speeds of participants and different scene lengths, an exact 70/30-split was not possible. Scenes: normal walking (nm-01, nm-02), carrying light object (ob-01), carrying heavy object (ob-02), chaotic background (ch-01), changes clothing (cl-01). Frames: total number of frames over all used IDs and scenes. Split: percentage based on frame count.

Phase	Data	Open-Set	Mixed-Set
Train	IDs	1 to 7	1 to 7
	Scenes	all	nm-01, nm-02, ob-01, ch-01
	Frames	68,055	54,132
	Split	≈70%	≈60%
Eval	IDs	8 to 10	1 to 7, 8 to 10
	Scenes	all	ob-02, cl-01
	Frames	26,304	34,090
	Split	≈30%	≈40%

**Table 2:** Keypoint displacement (in pixels) between original and anonymized frames (total frame count 22,665). Reported are the mean, median, and median absolute deviation (MAD) of frame pairs. Results show larger shifts (in bold) for joints farther from the body center and on the mostly occluded body side.

	Nose	Eye		Ear		Shoulder		Elbow		Wrist		Hip		Knee		Ankle	
Side		L	R	L	R	L	R	L	R	L	R	L	R	L	R	L	R
$\Delta_{\mathrm{mean}}$	3.5	3.2	3.3	3.7	4.6	5.8	6.5	**8.9**	**9.8**	**11.1**	**12.8**	5.3	6.3	6.8	7.6	**8.4**	**9.3**
$\Delta_{\mathrm{median}}$	2.6	2.5	2.4	2.4	2.5	2.5	2.4	3.4	**3.8**	**4.4**	**5.6**	3.2	3.4	3.4	**3.7**	**3.4**	**3.9**
$\Delta_{\mathrm{MAD}}$	1.2	1.2	1.2	1.3	1.5	1.4	1.4	**2.0**	**2.5**	**2.8**	**3.9**	1.7	1.9	2.0	**2.3**	2.0	**2.5**

## Data Availability

Data supporting the findings of this study are available from the authors upon reasonable request and declaration of intended use. The dataset cannot be made publicly available due to privacy and legal restrictions arising from recordings of identifiable individuals, as shown in this study.

## References

[1] Demertzi V., Demertzis S., Demertzis K. (2023). An Overview of Privacy Dimensions on the Industrial Internet of Things (IIoT). Algorithms.

[2] Ardabili B.R., Pazho A.D., Noghre G.A., Neff C., Ravindran A., Tabkhi H. (2022). Understanding ethics, privacy, and regulations in smart video surveillance for public safety. arXiv.

[3] Khan W., Topham L.K., Khayam U., Ortega-Martorell S., Panter H., Ansell D., Al-Jumeily D., Hussain A.J. (2024). Person de-identification: A comprehensive review of methods, datasets, applications, and ethical aspects along with new dimensions. IEEE Trans. Biometrics Behav. Identity Sci..

[4] Lin T.Y., Maire M., Belongie S., Bourdev L., Girshick R., Hays J., Perona P., Ramanan D., Zitnick C.L., Dollár P. Microsoft coco: Common objects in context. Proceedings of the 13th European Conference on Computer Vision (ECCV 2014).

[5] Hukkelas H., Lindseth F. DeepPrivacy2: Towards Realistic Full-Body Anonymization. Proceedings of the 2023 IEEE/CVF Winter Conference on Applications of Computer Vision (WACV).

[6] Jain A.K., Flynn P., Ross A.A. (2007). Handbook of Biometrics.

[7] Shen C., Yu S., Wang J., Huang G.Q., Wang L. (2025). A Comprehensive Survey on Deep Gait Recognition: Algorithms, Datasets, and Challenges. IEEE Trans. Biometrics Behav. Identity Sci..

[8] Filipi Gonçalves dos Santos C., Oliveira D.d.S., Passos L.A., Gonçalves Pires R., Felipe Silva Santos D., Pascotti Valem L., Moreira T.P., Santana M.C.S., Roder M., Paulo Papa J. (2022). Gait recognition based on deep learning: A survey. ACM Comput. Surv..

[9] Sepas-Moghaddam A., Etemad A. (2023). Deep gait recognition: A survey. IEEE Trans. Pattern Anal. Mach. Intell..

[10] Güner Şahan P., Şahin S., Kaya Gülağız F. (2024). A Survey of Appearance-Based Approaches for Human Gait Recognition: Techniques, Challenges, and Future Directions. J. Supercomput..

[11] Li T., Ma W., Zheng Y., Fan X., Yang G., Wang L., Li Z. (2024). A Survey on Gait Recognition against Occlusion: Taxonomy, Dataset and Methodology. PeerJ Comput. Sci..

[12] Cătrună A., Cosma A., Rădoi E. The paradox of motion: Evidence for spurious correlations in skeleton-based gait recognition models. Proceedings of the 2024 IEEE 18th International Conference on Automatic Face and Gesture Recognition (FG).

[13] Teepe T., Khan A., Gilg J., Herzog F., Hormann S., Rigoll G. Gaitgraph: Graph Convolutional Network for Skeleton-Based Gait Recognition. Proceedings of the 2021 IEEE International Conference on Image Processing (ICIP).

[14] Wu Z., Zhang C., Xu H., Jiao P., Wang H. (2025). DAGait: Generalized skeleton-guided data alignment for gait recognition. arXiv.

[15] Bashir K., Xiang T., Gong S. (2010). Gait Recognition without Subject Cooperation. Pattern Recognit. Lett..

[16] Van Mastrigt N.M., Celie K., Mieremet A.L., Ruifrok A.C.C., Geradts Z. (2018). Critical Review of the Use and Scientific Basis of Forensic Gait Analysis. Forensic Sci. Res..

[17] Yu S., Tan D., Tan T. A framework for evaluating the effect of view angle, clothing and carrying condition on gait recognition. Proceedings of the International Conference on Pattern Recognition.

[18] Nunes J.F., Moreira P.M., Tavares J.M.R. GRIDDS-a gait recognition image and depth dataset. Proceedings of the 6th International Conference on Computational Vision and Medical Image Processing (VipIMAGE 2019).

[19] Li W., Hou S., Zhang C., Cao C., Liu X., Huang Y., Zhao Y. An in-depth exploration of person re-identification and gait recognition in cloth-changing conditions. Proceedings of the IEEE/CVF Conference on Computer Vision and Pattern Recognition.

[20] Regulation (EU) 2016/679 of the European Parliament and of the Council of 27 April 2016 on the protection of natural persons with regard to the processing of personal data and on the free movement of such data, and repealing Directive 95/46/EC (General Data Protection Regulation) (Text with EEA relevance). https://eur-lex.europa.eu/eli/reg/2016/679/oj/eng.

[21] Government of Canada (2000). Personal Information Protection and Electronic Documents Act.

[22] Yoo J.L. (2011). Personal information protection in digital era—Reviewing Personal Information Protection Act. J. Digit. Converg..

[23] Abdulaziz S., Bondarev E. (2025). Unmasking performance gaps: A comparative study of human anonymization and its effects on video anomaly detection. arXiv.

[24] Jiang J., Skalli W., Siadat A., Gajny L. (2022). Effect of face blurring on human pose estimation: Ensuring subject privacy for medical and occupational health applications. Sensors.

[25] Zhang K., Luo W., Zhong Y., Ma L., Stenger B., Liu W., Li H. Deblurring by realistic blurring. Proceedings of the 2020 IEEE/CVF Conference on Computer Vision and Pattern Recognition (CVPR).

[26] Kupyn O., Martyniuk T., Wu J., Wang Z. Deblurgan-v2: Deblurring (orders-of-magnitude) faster and better. Proceedings of the IEEE/CVF International Conference on Computer Vision.

[27] Rozumnyi D., Oswald M.R., Ferrari V., Matas J., Pollefeys M. DeFMO: Deblurring and Shape Recovery of Fast Moving Objects. Proceedings of the IEEE/CVF Conference on Computer Vision and Pattern Recognition.

[28] Ban B., Lee H. (2024). FAKER: Full-body anonymization with human keypoint extraction for real-time video deidentification. arXiv.

[29] Brkic K., Sikiric I., Hrkac T., Kalafatic Z. I Know That Person: Generative Full Body and Face De-identification of People in Images. Proceedings of the 2017 IEEE Conference on Computer Vision and Pattern Recognition Workshops (CVPRW).

[30] Zwick P., Roesch K., Klemp M., Bringmann O. (2024). Context-aware full body anonymization using text-to-image diffusion models. arXiv.

[31] Hukkelås H., Lindseth F. Does image anonymization impact computer vision training?. Proceedings of the 2023 IEEE/CVF Conference on Computer Vision and Pattern Recognition (CVPR).

[32] Triess S.C., Leitritz T., Jauch C. Exploring AI-based Anonymization of Industrial Image and Video Data in the Context of Feature Preservation. Proceedings of the 2024 32nd European Signal Processing Conference (EUSIPCO).

[33] Lee J.H., You S.J. (2024). Balancing Privacy and Accuracy: Exploring the Impact of Data Anonymization on Deep Learning Models in Computer Vision. IEEE Access.

[34] Zhou J., Beyerer J. Impacts of data anonymization on semantic segmentation. Proceedings of the 2022 IEEE Intelligent Vehicles Symposium (IV).

[35] Weiß S., Bonenberger C., Niedermaier T., Knof M., Stähle B., Schneider M. Towards Systematic Evaluation of Computer Vision Models Under Data Anonymization. Proceedings of the 48th German Conference on Artificial Intelligence (Künstliche Intelligenz).

[36] Romero D., Patel R.J., Markopoulou A., Elmalaki S. (2025). GaitGuard: Protecting Video-Based Gait Privacy in Mixed Reality. arXiv.

[37] Meng S., Fu Y., Hou S., Cao C., Liu X., Huang Y. (2023). Fastposegait: A toolbox and benchmark for efficient pose-based gait recognition. arXiv.

[38] Meng S., Fu Y., Hou S., Hu X., Cao C., Liu X., Huang Y. (2025). From FastPoseGait to GPGait++: Bridging the Past and Future for Pose-Based Gait Recognition. IEEE Trans. Pattern Anal. Mach. Intell..

[39] Teepe T., Gilg J., Herzog F., Hormann S., Rigoll G. Towards a Deeper Understanding of Skeleton-based Gait Recognition. Proceedings of the 2022 IEEE/CVF Conference on Computer Vision and Pattern Recognition Workshops (CVPRW).

[40] Zhang C., Chen X.P., Han G.Q., Liu X.J. (2023). Spatial transformer network on skeleton-based gait recognition. Expert Syst..

[41] Fu Y., Meng S., Hou S., Hu X., Huang Y. GPGait: Generalized Pose-based Gait Recognition. Proceedings of the 2023 IEEE/CVF International Conference on Computer Vision (ICCV).

[42] Fu Y., Hou S., Meng S., Hu X., Cao C., Liu X., Huang Y. Cut Out the Middleman: Revisiting Pose-Based Gait Recognition. Proceedings of the 18th European Conference on Computer Vision (ECCV 2024).

[43] Oshita K. (2021). Immediate after-effects of shapes of clothing worn on tandem gait performance. Acta Bioeng. Biomech..

